# A Phase III, Randomized, Non-Inferiority Trial to Assess the Efficacy and Safety of Dihydroartemisinin-Piperaquine in Comparison with Artesunate-Mefloquine in Patients with Uncomplicated *Plasmodium falciparum* Malaria in Southern Laos

**DOI:** 10.4269/ajtmh.2010.10-0276

**Published:** 2010-12-06

**Authors:** Mayfong Mayxay, Sommay Keomany, Maniphone Khanthavong, Phoutthalavanh Souvannasing, Kasia Stepniewska, Tiengthong Khomthilath, Siamphay Keola, Tiengkham Pongvongsa, Samlane Phompida, David Ubben, Neena Valecha, Nicholas J. White, Paul N. Newton

**Affiliations:** Wellcome Trust, Mahosot Hospital, Oxford University Tropical Medicine Research Collaboration, Mahosot Hospital, Vientiane, Laos; Faculty of Postgraduate Studies, University of Health Sciences, Vientiane, Laos; Salavan Provincial Hospital, Salavan Province, Laos; Centre of Malariology, Parasitology and Entomology, Vientiane, Laos; Phalanxay District Clinic, Savannakhet Province, Laos; Savannakhet Provincial Malaria Station, Savannakhet Province, Laos; Medicines for Malaria Venture, Geneva, Switzerland; Centre for Clinical Vaccinology and Tropical Medicine, Churchill Hospital, University of Oxford, Oxford, United Kingdom; Faculty of Tropical Medicine, Mahidol University, Bangkok, Thailand; National Institute of Malaria Research, Delhi, India

## Abstract

We conducted an open, randomized clinical trial of oral dihydroartemisinin-piperaquine (DP) versus artesunate-mefloquine (AM) in 300 patients in Laos with uncomplicated *Plasmodium falciparum* malaria as part of a multicentre study in Asia. Survival analysis and adjustment for re-infection showed that the 63-day cure rates (95% confidence interval [CI]) were 100% for AM and 99.5% (96.4–99.8%) for DP. The 63-day cure rates per protocol were 99% (97 of 98) for AM and 99.5% (196 of 197) for DP (*P* = 0.55). The difference (AM minus DP) in cure rates (95% CI) was −0.5% (−5.1 to 2.0%), which is within the 5% non-inferiority margin. The median fever and parasite clearance times were also similar for AM and DP. The proportion of patients with at least one recorded potential adverse event was significantly higher in the AM group (38 of 87, 44%) than in the DP group (57 of 182, 31%) (relative risk = 0.6, 95% CI = 0.4–0.9; *P* = 0.04). Dihydroartemisinin-piperaquine is not inferior to AM in the treatment of uncomplicated *P. falciparum* malaria in Laos and is associated with fewer adverse effects. The results of this study were similar to those of the larger multicentre study.

## Introduction

Malaria remains an important public health challenge in southern Laos with a median incidence of *Plasmodium falciparum* infection of 4.7–23.5/1,000 population.^1^ As in many other tropical countries, antimalarial drug resistance to *P. falciparum* poses a public health threat. The Lao Government changed national policy for first-line antimalarial drug treatment of uncomplicated *P. falciparum* malaria to artemether-lumefantrine (AL) in 2005. This artemisinin-combination treatment (ACT) and artesunate-mefloquine both have high efficacies and good tolerability with 42-day failure rates of ≤ 6% in Laos.^2^,^3^ However, there is uncertainty as to the clinical importance of reduced bioavailability of lumefantrine when taken without fatty food,^4^–^8^ especially with evidence of resistance to artemisinin derivatives in adjacent Cambodia increasing the required contribution to efficacy by the partner drug.^9^,^10^ Artemether-lumefantrine has to be taken twice a day, which reduces adherence.

Dihydroartemisinin-piperaquine (DP) is a potential alternative; it can be taken once a day, does not have food-dependent bioavailability, and can be produced for a lower cost than AL. A trial comparing DP (Artekin^™^; Holleykin Pharmaceutical Co., Guangzhou, China) with artesunate (Guilin Pharmaceutical Co., Guilin, China) plus mefloquine (Lariam^™^; Roche, Basel, Switzerland) in southern Laos demonstrated that DP had a 100% cure rate (n = 110) assessed at 42 days-follow up.^11^ The DP used in this study was produced in compliance with Chinese Good Manufacturing Practice (GMP) standards, but not with the GMP standards required by drug regulatory authorities in Europe or the United States. A DP formulation produced to standards compliant with International Conference on Harmonization GMP has been developed by Sigma-Tau (Rome, Italy) with support from the Medicines for Malaria Venture (MMV).

We therefore conducted a phase III, randomized, non-inferiority trial of this new formulation to assess the efficacy and safety in comparison with artesunate-mefloquine (AM) in patients with acute, uncomplicated *P. falciparum* malaria in Laos. This study was part of a multi-center study in Asia that included India, Laos, and Thailand.^12^ This paper reports the detailed results from Laos and their implications.

## Materials and Methods

### Study site, patients, clinical procedures, and laboratory investigation.

The study was conducted during June–October in 2005 and 2006 at Phalanxay (10 beds) and Xepon (30 beds) District Clinics and during June–October 2006 at Xepon District Clinic, Savannakhet Province. Phalanxay and Xepon districts (Savannakhet Province) are located 605 km and 665 km, respectively, southeast of Vientiane, the capital of Laos. Phalanxay (78 villages, population = 24,730) and Xepon (88 villages, population = 48,000) are inhabited predominantly by rice farmers of the Lao Theung ethnic group.^2^,^11^,^13^,^14^ A sample size calculation was performed for the whole multi-center study. For Laos, 300 patients were assigned to be recruited.^12^

Patient inclusion and exclusion criteria have been reported.^12^ At presentation, venous blood samples were obtained for parasite count, hematologic tests, and biochemical tests; three blood spots were collected on 3MM filter paper (Whatman, Maidstone, United Kingdom) for genotyping by polymerase chain reaction (PCR) in the event of reappearance of *P. falciparum* during follow-up.^13^ A 12-lead electrocardiogram (ECG) (CarTouch; Cardionics S.A., Belgium) was obtained.

If the study inclusion criteria were met, the patients were randomly assigned to receive either 1) AM: artesunate (Artesunate, 100 oblong Lactab; Mepha, Aesch-Basel, Switzerland), 4 mg/kg/day for 3 days (days 0–2) plus mefloquine (Mephaquin Lactab; Mepha, Aesch-Basel), 15 mg base/kg on day 1 and 10 mg base/kg on day 2 or 2) DP: dihydroartemisinin-piperaquine (Sigma-Tau), 2.1/16.8 mg/kg in a single daily dose for 3 days. Each tablet contained 40 mg of dihydroartemisinin and 320 mg of piperaquine for adults and 20 mg of dihydroartemisinin and 160 mg of piperaquine for children < 15 years of age. The use of AM and DP in this study was reviewed and approved by the Food and Drug Department, Ministry of Health, Laos.

The treatment choice was kept in a sealed opaque envelope, which was opened only after the decision to recruit had been made. An unequal randomization, 2:1 (DP:AM), was used to provide more precise estimates of DP cure rates and to provide more patients for the safety database of DP. Axillary temperature was measured every six hours. Study drug administration was observed directly by the study physicians. Study medications administered to older children (> 5 years of age) and adults were given as tablets or fractions of tablets orally with a glass of water and those given to young children (≤ 5 years of age) were crushed, mixed with water, and administered as a slurry if the children were unable to swallow. Patients were observed for one hour to ensure that the medications were not vomited or regurgitated.^12^

Patients were reviewed daily until parasite clearance was observed, then weekly for 63 days from the start of treatment, or at other times if he or she was ill. At each visit, finger prick blood was obtained for a malaria blood smear and hematocrit. Three blood spots were collected on filter paper from those with reappearance of asexual parasitemia for genotyping.^15^ Twelve-lead ECGs were obtained on days 2 and 7 and on days 28 and 63 if the ECG result was abnormal on day 7; ECGs were also obtained on the day of *P. falciparum* recurrence. Venous blood samples were obtained for hematologic and biochemical tests on days 28 and 63 if the results were abnormal on day 28 and were also performed on the day of *P. falciparum* recurrence.

Patients who received AM and had recurrent *P. falciparum* parasitemia or treatment failure were withdrawn from the study, re-treated with oral artesunate (2 mg/kg/day) plus doxycycline (4 mg/kg/day) (or artesunate alone for children < 8 years of age) for 7 days and followed-up. Those persons who received DP and had recurrent *P. falciparum* parasitemia or treatment failure were withdrawn from the study, re-treated with AM for three days, and followed-up. Patients in whom severe disease developed^16^ or required rescue therapy were re-treated with intravenous artesunate, 2.4 mg/kg/day initially, and then oral or intravenous drug to give a total treatment course of 12 mg/kg over 7 days. Those persons who received additional courses of therapy were also followed-up for 63 days. Potential adverse events were recorded for those ≥ 3 years of age and able to answer questions about these symptoms. Written informed consent was obtained from all adult participants and from parents or legal guardians of minors. Ethical clearance for the study was obtained from the Lao National Ethics Committee for Health Research and the Oxford University Tropical Medicine Research Ethics Committee, United Kingdom. The trial registration number is ISRCTN81306618. The trial was monitored by MDS Pharma Services (http://www.mdsps.com/).

Parasite counts in thick and thin blood films stained with Field's stain were obtained daily until parasite clearance and then weekly from day 7.^13^ Polymerase chain reaction amplification was performed on paired samples for parasite genotyping to distinguish between reinfection and recrudescence.^15^

### Outcome measures.

The primary end point was the PCR-corrected adequate clinical and parasitologic response PCR-ACPR at day 63 by using World Health Organization (2003) guidelines.^17^ Analysis was performed for each patient until either day 63 or until the reappearance of *P. falciparum* parasites.

Secondary end points were 1) crude or PCR-uncorrected adequate clinical and parasitologic response, 2) proportion of patients with treatment failure at or after day 7, 3) proportion of aparasitemic patients on days 1, 2, and 3, 4) parasite clearance time (time in days from first treatment dose to the first thick blood film negative for *P. falciparum* parasites after checking ≥ 500 oil-immersion microscopic fields), 5) proportion of a febrile patients on days 1, 2, and 3, 6) fever clearance time (time in hours from the start of treatment at which the axillary temperature first decreased below 37.5°C and remained below 37.5°C for 48 hours), 7) gametocyte carriage (blood slide positive for gametocytes) after treatment, 8) fractional changes in hematocrit after antimalarial treatment, and 9) adverse events.

### Statistical analysis.

Data were analyzed by using SPSS version 11.0 (SPSS, Chicago, IL). Comparisons between two groups were made by using the Mann-Whitney U test, the Student's *t*-test, the chi-square test, and Fisher's exact tests, as appropriate. Cure rates were calculated as the proportion of patients with PCR-confirmed recrudescence by using intention-to-treat (ITT) and per-protocol (PP) populations.^17^ In the ITT population, all losses to follow-up were treated as failures. In the PP population, losses to follow-up were excluded from the analysis. Patients with new infections were regarded as cures in both analyses. Survival analysis (Kaplan-Meier estimates) was used to calculate cure rates by using Stata version 9 (StataCorp, College Station, TX) for all randomized patients.^18^ Patients with new infections were censored at the time of the new infection, and patients lost to follow-up were censored at their last visit. Confidence intervals (CIs) for the difference in cure rates calculated for the PP population were estimated by using Newcombe's method and were used to test the non-inferiority hypothesis.^19^

Gametocyte carriage was summarized as person-gametocytes-week rates calculated as the total number of weeks with gametocytes divided by the total number of weeks of follow-up and presented as per 1,000 person-weeks.

## Results

**Figure 1.**
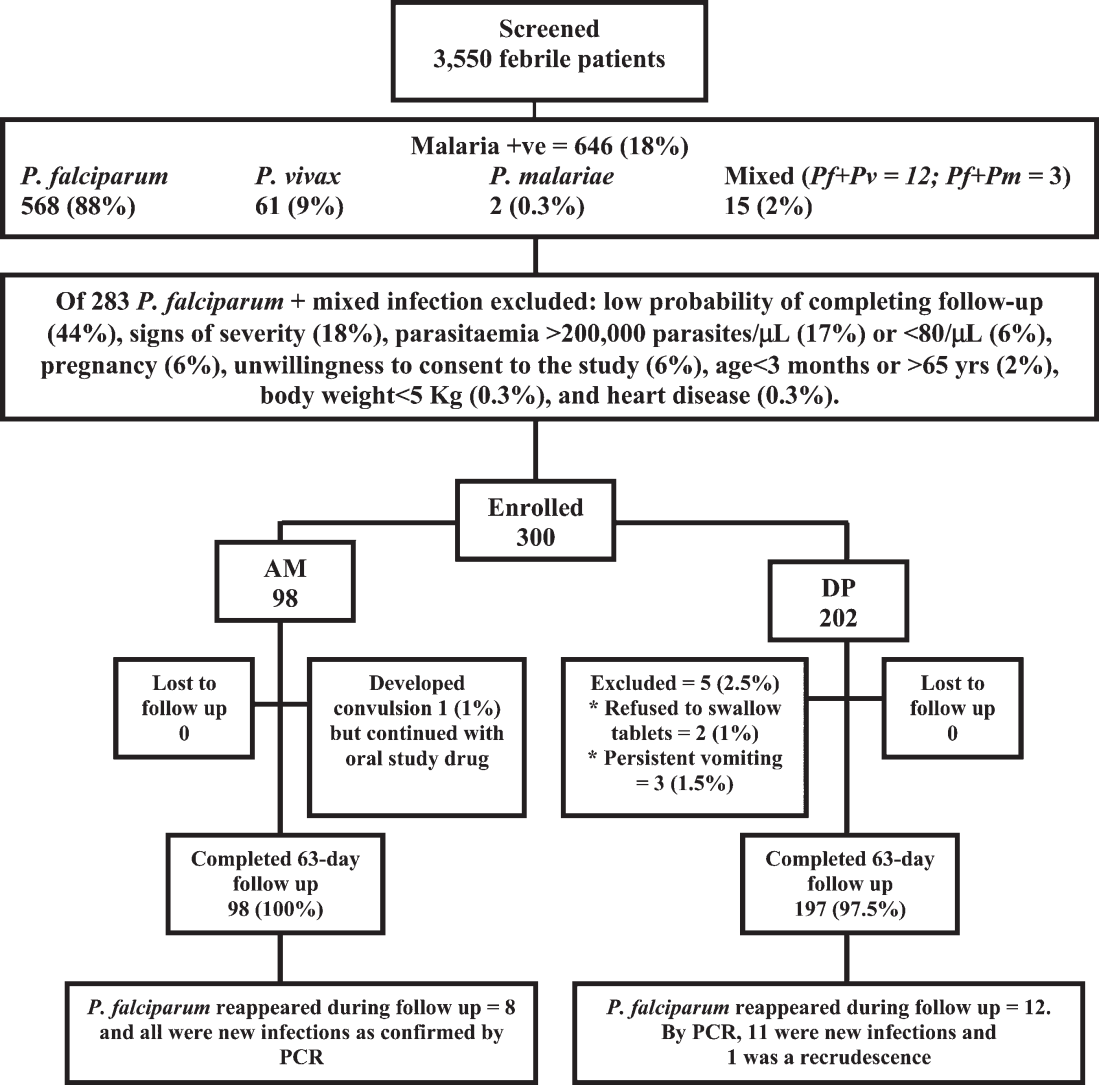
Patient flow diagram. +ve = positive; *Pf* = *Plasmodium falciparum*; *Pv* = *P. vivax*; *Pm* = *P*. *malariae*; AM = artesunate-mefloquine; DP = dihydroartemisinin-piperaquine; PCR = polymerase chain reaction.

The numbers of patients screened for malaria and enrolled in the trial is shown in Figure 1. The admission features of the two study groups were similar (Table 1). At the time of enrollment, 129 (43%) patients were afebrile but had a history of fever prior to admission. The proportion of patients without documented fever on admission was similar in the AM and DP groups. Most patients (68%, 205 of 300) were children (≤ 15 years of age) (69 of 98 in AM group and 136 of 202 in the DP group). Two patients in the DP group refused to swallow the study tablets, and three patients had persistent vomiting after taking study drugs, and thus were excluded from the study and were not followed-up. Therefore, 98 (100%) and 197 (97.5%) patients in AM and DP groups, respectively, completed 63 days of follow-up (Figure 1).

### Cure rates, fever and parasite clearance, and changes in hematocrit and hemoglobin levels.

Severe disease developed in a four-year-old girl who had uncomplicated *P. falciparum* malaria (97,968 parasites/μL). She had one 5-minute convulsion 30 hours after receiving AM and remained unconscious (Glasgow Coma Score ≥ 9/15) for 70 minutes. She recovered after receiving supportive treatment. This patient was not excluded from the study and completed 63 days of follow-up and showed cure.

Of 8 and 12 patients with subsequent *P. falciparum* reappearance in the AM and DP groups, respectively, PCR analysis indicated that only one patient, in the DP group, had a recrudescent infection. If we considered the four-year-old patient with the convulsion and coma after treatment as an early treatment failure, the 63-day cure rates per protocol, excluding patients who refused to swallow tablets or had persistent vomiting, or experienced re-infection, were 99% (97 of 98) for AM and 196 of 197 (99.5%) for DP. The difference (AM minus DP) in cure rates (95% CI) of −0.5% (−5.1 to 2.0%) is within the 5% non-inferiority margin. After censoring the patient who had convulsions, those experiencing re-infections and those who refused to swallow the study drugs or had repeated vomiting, the cure rates (95% CI) by survival analysis (Kaplan-Meier) were 100% for AM and 99.5% (96.4–99.8%) for DP (Table 2). If the four-year-old girl with severe disease was considered an early treatment failure, the cure rates (95% CI) were 99.0% (93.0–99.9%) and 99.5% (96.4–99.9%) for the AM and DP groups, respectively. Conventional intention-to-treat analysis showed that the 63-day cure rates adjusted for re-infection were 99% (97 of 98) for AM and 97% (196 of 202) for DP (*P* = 0.43). Similarly, 28-day cure rates by intention-to-treat analysis were 99% (97 of 99) for AM and 97.5% (197 of 202) for DP. Intention-to-treat and per protocol analyses showed that the 63-day PCR-uncorrected cure rates for AM and DP were 89 of 98 (91%) versus 190 of 202 (94%) (*P* = 0.16), and 89 of 91 (91%) versus 185 of 197 (94%) (*P* = 0.33), respectively. The number of patients with *P. vivax* appearance in the AM and DP groups during follow-up was similar (Table 2).

At presentation, 57% of all patients had fever, as defined by a body temperature ≥ 37.5°C. At day 2, 99% and 97.5% in AM and DP groups, respectively, were afebrile (*P* = 0.67), and all patients (except one in the DP group) had no parasites detected. The median fever and parasite clearance times were similar between the AM and DP groups (Table 2). Comparison of baseline (day 0) and day 28 mean hematocrits showed that anemia improved significantly after treatment in the AM and DP groups (*P* < 0.001 for both, by paired *t*-test) (Table 2). The mean hematocrit on day 7 was significantly higher in the DP group than in the AM group (*P* = 002). Mean leukocyte counts were significantly higher on day 28 than on day 0 in both treatment groups (*P* < 0.001).

### Comparisons between adults and children.

The mean admission temperature was significantly higher in children (38.0°C [37.8–38.1°C]) than adults (37.7°C [37.4–37.9°C]) (*P* = 0.04). A palpable spleen (47% and 4%; *P* < 0.001) and liver (25% and 2%, *P* < 0.001) were significantly more frequent in children than adults. The admission geometric mean parasitemia/μL was significantly higher in children than adults (13,718 parasites/μL [10,578–17,795 parasites/μL) versus 6,471 parasites/μL [4,578–9,147 parasites/μL]) (*P* = 0.001), as were the proportions of patients with clinical anemia and vomiting at presentation (41 [20%] of 205 versus 1 [1%] of 95; *P* < 0.001 and 94 [46%] of 205 versus 29 [30.5%] of 95; *P* = 0.01; respectively). The frequency of the following admission symptoms and signs were significantly lower in children than adults: weakness (154 [75%] of 205 versus 85 [89%] of 95; *P* = 0.004), muscle pain (32 [15%] of 205 versus 80 [84%] of 95; *P* < 0.001), headache (144 [70%] of 205 versus 91 [96%] of 95; *P* < 0.001), nausea (76 [37%] of 205 versus 48 [50.0%] of 95; *P* = 0.02), pruritis (0% versus 3 [3%] of 95; *P* < 0.03), and tinnitus (3 [1%] of 205 versus 15 [16%] of 95; *P* < 0.001).

The median and range fever and parasite clearance times were not significantly different between children (23 [7–64] hours and 2 [1–3] days) and adults (21 [7–68] hours and 2 [1–2] days) (*P* = 0.48 and 0.06, respectively, by Mann-Whitney U test). Mean hematocrit values on admission and on all days between days 7 and 63 were significantly lower in children than in adults (*P* < 0.001) in both treatment groups.

#### *Plasmodium falciparum* gametocyte carriage.

Twenty-three (7.7%) patients had patent gametocytemias on admission, 6 (6%) in the AM group and 17 (8%) in the DP group (*P* = 0.48). The time to clearance of gametocytes was longer in the DP group than in the AM group. All patients in the AM group and only 8 (47%) patients in the DP group cleared their gametocytemias by day 7 (*P* = 0.048, by Fisher's exact test). After treatment, gametocytemia developed on day 56 in one patient in the AM group who did not have gametocytemia on admission at the same time as a new *P. falciparum* infection. Gametocytemia also developed after admission (days 1–2), which cleared by day 14, in 6 other patients in the DP group who did not have gametocytemia on admission. The proportion of patients in whom gametocytemia developed in the AM and DP groups was not significantly different (*P* = 0.68). Person-gametocyte-weeks was estimated as 12 weeks/1,000 patients in the AM group and 22 weeks/1,000 patients in the DP group (*P* = 0.069). The proportion of patients with gametocytemia at any time point was higher in children (13%, 27 of 205) than in adults (3%, 3 of 95) (*P* = 0.006, by Fisher's exact test), and the proportion of patients with gametocytemia after treatment was 4% (7 of 185) in children and 0% (0 of 92) in adults (*P* = 0.10, by Fisher's exact test).

### Adverse events.

Adverse events were reported by 269 (89.7%) patients ≥ 3 years of age who were able to answer questions about these symptoms. A convulsion and coma developed in a four-year-old patient after treatment with AM (see above). Severe malaria or other life-threatening events after treatment did not develop in any other patients. The frequency of patients with symptoms and signs before treatment that may subsequently be confused with drug-related adverse events did not differ significantly between groups (*P* > 0.05). The proportion of patients with at least one recorded potential side effect/adverse event after treatment was significantly higher in the AM group (38 of 87, 44%) than in the DP group (57 of 182, 31%) (*P* = 0.04). The incidence of post-treatment dizziness, nausea, insomnia, and anorexia were all significantly higher in AM group than in the DP group (*P* ≤ 0.01) (Table 3). The frequency of some adverse events was significantly higher in adults than in children (see above).

### Electrocardiograms.

No significant ECG abnormalities were found before or after treatment. The mean ECG-determined heart rate was significantly lower after treatment at days 2 and 7 for both treatment groups (*P* < 0.001) than at admission (Table 4). The mean PR interval was significantly longer after treatment (on days 2 and 7) than on admission for both groups (*P* ≤ 0.001): mean PR interval (95% CI) difference between baseline and days 2 and 7 were −9.5 (−13.1 to −6.0) and −5.7 (−9.1 to −2.4), respectively, for the AM group and −5.3 (−7.1 to −3.5) and −3.6 (−5.6 to −1.6), respectively, for the DP group. Four patients (two in the AM group and two in the DP group) had PR interval prolongation (> 200 ms) after treatment on day 2 or day 7 but the PR intervals on day 7 or day 28 were normal. The mean QRS interval was also significantly longer on day 2 than on admission (*P* = 0.001) but was similar to the baseline value by day 7 (*P* > 0.05) in both treatment groups (Table 4).

Modeling the QT/RR relationship^20^–^22^ gave a correction formula of QR/RR0.521 pre-drug, QR/RR0.478 on day 2, and QR/RR0.493 on day 7. Because this calculation approximates to Bazzet's formula (QTc = QT/RR × 0.5), Bazzet's correction was used. There was weak negative correlation or no correlation between QTc and RR intervals at baseline or after treatment (Table 4). The mean (95% CI) QTc (ms) was significantly prolonged on day 2 than at baseline in the DP group (*P* < 0.001) (Table 4). The mean (95% CI) QTc (ms) prolongation on days 2 and 7 compared with those at baseline were 3.5 (−1.1 to 8.2) and 5.2 (−0.9 to 11.4), respectively, for the AM group and 9.5 (5.9–13.1) and 1.4 (−2.5 to 5.4), respectively, for the DP group. The proportion of the patients with prolonged QTc pre-treatment and on days 2 and 7 did not differ significantly between the AM and DP groups (*P* > 0.05) (Table 4). The numbers of patients with QTc lengthening (defined as > 30 ms and > 60 ms by the U.S. Food and Drug Administration^23^,^24^) were not significantly different between the two treatment groups (*P* > 0.05) (Table 4).

## Discussion

This study confirms that the fixed-dose, co-formulated ACT dihydroartemisinin-piperaquine is not inferior to the more expensive, not co-formulated AM in the treatment of uncomplicated *P. falciparum* malaria in southern Laos and showed a 63-day follow-up cure rate of almost 100% for both drugs. Our previous study conducted in the same area, using GMP drugs from China, also demonstrated that the 42-day follow up cure rates were 99% and 100% in AM and DP groups, respectively.^11^ The present study was part of a large multi-center trial in Asia that enrolled 1,150 patients.^12^ The overall day 63 cure rates at all sites were 97.0% for the DP group and 95.3% for the AM group in the modified ITT population and 98.7% for the DP group and 97.0% for the AM group in the per protocol population, which exceed the World Health Organization acceptance threshold for day 63 PCR-corrected cure rates of 95%.

The 26 published clinical trials conducted in Asia, Africa, and South America that compared the efficacy of DP with other ACT regimens (Table 5) show cure rates after treatment with DP, regardless of duration of follow up, of > 95% in 22 of the 26 studies. In a meta-analysis of individual patient data from 7 trials in Africa and Southeast Asia that included 1,814 patients with uncomplicated *P. falciparum* malaria who were treated with DP, the 63 day PCR-corrected cure rate was 97.7%.^53^

An important limitation of this trial for public health policy in Laos is that DP was compared with AM and not with AL, which is the current Lao national treatment policy. Artesunate-mefloquine was the comparator because this was required for the multicentre trial. In 2005, AM was the intended national treatment policy but sudden and unexpected unavailability of AM led the Government of Laos to change to AL. Artesunate-mefloquine is national policy in Cambodia, Myanmar (Burma), and Thailand, and DP is national policy in Vietnam and China. There have been six comparisons of DP versus AL, all in children from Africa, and the 28–63-day cure rates were not significantly different between AL (84–96%) and DP (93–100%).^25^–^30^

In the multi-center study, overall *P. falciparum* gametocyte prevalence (days 0–70) measured as person-gametocyte-weeks was significantly higher after DP than AM (9.7% versus 4.8%; *P* = 0.006; modified ITT).^12^ In addition, Zwang and others^53^ found that the proportion of patients in whom gametocytemia developed and the gametocyte carriage rate was higher after DP than after the comparator. However, in Laos, the proportion of patients with gametocytemia at any time point on or after day 7 and the mean (95% CI) person-gametocyte-weeks did not significantly differ between the AM and DP groups, probably because of the smaller sample size.

Of 26 published clinical trials of either DP or DP plus trimethoprim (Table 5) that included 6,010 patients, 8 deaths were reported (6 children and 2 adults). One adult death was probably caused by severe malaria that developed during treatment with DP; the other seven deaths were considered unlikely to be a consequence of DP. No deaths were reported in the larger multi-center trial of DP in Asia of 767 patients, of which this trial was a part.^12^

Although piperaquine has a chemical structure similar to chloroquine, which can cause lethal cardiologic effects,^31^ results of our study are consistent with that of previous studies, which suggested that at therapeutic doses, DP does not have clinically significant effects on the ECG results.^32^–^34^

In Laos, AL is provided by the Global Fund at a cost of < 1.4 U.S.$/adult treatment course and is free to patients through the public sector. When compared with AL, DP has potential benefits because it is less expensive to manufacture, may have better adherence, and can be used in a once a day regimen rather than a twice a day regimen. Although a study of the unsupervised effectiveness and supervised efficacy of DP and AM treatment in western Myanmar suggested good adherence to both regimens, there have been no studies directly comparing DP and AL adherence.^35^ Although DP has been successfully used as rescue therapy for multidrug-resistant *P. falciparum* malaria in pregnancy (PCR-adjusted cure rate at 63 days was 92%),^36^ further studies are needed to establish its safety in pregnancy.

In conclusion, DP demonstrated non-inferiority to the reference treatment of AM in Laos and in the overall three-country study. The high efficacy and relative safety of DP and its availability as a fixed combination and probable lower cost compared with other candidate ACTs suggests that it could play a significant role in the first-line treatment of uncomplicated *P. falciparum* malaria in Laos.

## Figures and Tables

**Table 1:** Admission demographic, clinical, and laboratory details for patients in a study comparing AM and DP for treatment of *Plasmodium falciparum* malaria in Laos*

Variable	Treatment groups		
	All (n = 300)	AM (n = 98)	DP (n = 202)
Sex, Male, no. (%)	179 (60)	59 (60)	120 (59)
Age, years	14.4 (12.8–16.0)	14.1 (11.2–17.0)	14.6 (12.7–16.5)
Body weight, kg	29.3 (27.2–31.4)	28.2 (24.5–31.9)	29.8 (27.2–32.4)
Previous malaria attack, no. (%) of patients†	99 (33)	34 (35)	65 (32)
Axillary temperature, °C	37.9 (37.7–38.0)	37.9 (37.7–38.2)	37.9 (37.7–38.0)
Patients without fever on admission, no. (%)	129 (43)	39 (40)	90 (44.5)
Systolic blood pressure, mm Hg‡	103.3 (101.8–104.9)	104.5 (101.6–107.4)	102.8 (100.9–104.6)
Diastolic blood pressure, mm Hg‡	64.5 (63.3–65.7)	65.0 (62.8–67.3)	64.2 (62.8–65.7)
Pulse, beats/min	109.6 (107.1–112.0)	110.2 (105.6–114.9)	109.2 (106.3–112.2)
Splenomegaly, no. (%) of patients	100 (33)	37 (38)	63 (31)
Hepatomegaly, no. (%) of patients	54 (18)	21 (21)	33 (16)
Parasitemia, geometric mean parasites/μL	20,564 (17,873–23,659)	22,851 (18,433–28,333)	18,505 (15,438–22,182)
Erythrocytes/mm^3^	4.91 (4.81–5.02)	4.79 (4.62–4.96)	4.97 (4.85–5.10)
Hemoglobin, g/dL	11.4 (11.1–11.7)	11.2 (10.7–11.7)	11.5 (11.1–11.8)
Hematocrit, %	36.6 (35.8–37.4)	35.9 (34.6–37.3)	36.9 (35.9–38.0)
Leukocytes/mm^3^	6,679 (6,322–7,035)	6,754 (6,237–7,270)	6,642 (6,173–7,112)
PMN, %	61.8 (60.4–63.2)	63.6 (61.2–65.9)	60.9 (59.2–62.7)
Lymphocytes, %	34.5 (33.2–35.8)	33.4 (31.2–35.7)	35.1 (33.4–36.7)
Platelets/mm^3^	108,010 (100,44–115,536)	109,375 (96,369–122,380)	107,351 (98,050–116,653)
ALT, IU/L, median (range)	24 (6–272)	23 (7–131)	25 (6–272)
AST, IU/L, median (range)	33 (12–361)	32 (14–244)	33 (12–361)
GGT, IU/L, median (range)	15 (6–584)	15 (7–584)	15 (6–238)
Alkaline phosphatase, IU/L	149.6 (141.7–157.4)	152.2 (139.8–164.5)	148.3 (138.2–158.3)
Total bilirubin, μmol/L	16.7 (15.4–17.9)	18.3 (15.6–21.2)	15.9 (14.5–17.1)
Direct bilirubin, μmol/L	4.3 (3.9–4.8)	4.8 (3.9–5.5)	4.1 (3.6–4.6)
Total protein, g/L	68.5 (67.6–69.3)	68.4 (67.0–69.7)	68.5 (67.5–69.5)
Blood urea nitrogen, mmol/L	4.6 (4.3–4.8)	4.8 (4.3–5.2)	4.5 (4.2–4.8)
Creatinine, μmol/L	48.6 (46.0–51.3)	46.8 (42.4–45.2)	48.6 (46.0–52.2)
Glucose, mmol/L	5.9 (5.7–6.1)	6.1 (5.6–6.6)	5.8 (5.6–6.0)
Albumin, g/L	35.5 (35.0–36.0)	36.0 (35.0–37.0)	35.0 (34.0–36.0)

* Values are presented as mean (95% confidence intervals) unless otherwise indicated. AM = mefloquine plus artesunate for 3 days; DP = dihydroartemisinin plus piperaquine for 3 days; PMN = polymorphonuclear leukocytes; ALT = alanine aminotransferase; AST = aspartate aminotransferase, GGT = γ-glutamyl transferase.

† Defined as patient or patient's guardian reporting that the patient had had a febrile illness with a positive malaria slide.

‡ Data were available from only 201 patients in DP group

**Table 2:** Outcome measures for the treatment of patients enrolled in a study comparing AM and DP for treatment of *Plasmodium falciparum* malaria in Laos*

Variable	Treatment groups		
	All (n = 300)	AM (n = 98)	DP (n = 202)
63-day cure rate, no. (%) of patients†	–	97/98 (99)	196/202 (97)
63-day cure rate per protocol, no. (%) of patients	–	97/98 (99%)	196/197 (99.5)
63-day cure rate by survival analysis, % (95% CI)	–	100	99.5 (96.4–99.8)
Fever clearance time, median hours (range)†‡	22 (7–68)	24 (7–68)	21 (7–64)
Parasite clearance time, median days (range)†§	2 (1–3)	2 (1–2)	2 (1–3)
Positive parasitemia at day 1, no. (%) of patients	199/298 (67)	70/98 (71)	129/200 (64.5)
Positive parasitemia at day 2, no. (%) of patients	1/298 (0.3)	0	1/200 (0.5%)
Gametocytemia after treatment, no. (%) of patients	7/300 (2.3)	1/98 (1)	6/202 (3)
*P. vivax* appearance after treatment of *P. falciparum*, no. (%) of patients	10/295 (3)	2/98 (6)	4/197 (2)
Day 0 hematocrit, mean % (95% CI)	36.6 (35.8–37.4)¶	35.9 (34.6–37.3) ¶	36.9 (35.9–38.0) ¶
Day 7 hematocrit, mean % (95% CI)#	32.6 (32.0–33.3)	31.6 (30.5–32.7)	33.1 (32.4–34.0)
Day 28 hematocrit, mean % (95% CI)	38.6 (38.2–39.1)	38.2 (37.4–39.0)	38.8 (38.2–39.5)

* AM = mefloquine plus artesunate for 3 days; DP = dihydroartemisinin plus piperaquine for 3 days; CI = confidence interval.

† Intention-to-treat analysis.

‡ Data were available from only 81 and 151 patients in the AM and DP groups, respectively.

§ Data were available from only 98 and 197 patients in the AM and DP groups, respectively.

¶ Significant difference from day 28 (*P* < 0.01).

# Significant difference between AM and DP groups (*P* = 0.02).

**Table 3:** Possible adverse events in patients in a study comparing AM and DP for treatment of *Plasmodium falciparum* malaria in Laos*

Sign or symptom	Prior to treatment (n = 300)	After treatment		
		AM (n = 98)	DP (n = 202)	*P*
At least one AE†	95 (35)	38 (44)	57 (31)	0.04
Cardiologic AE‡	61 (20)	16 (16)	45 (22)	0.23
T-wave abnormality	1 (0.3)	0	1 (0.5)	1.00
Complete bundle block§	2 (0.7)	2 (2)	0	0.10
Prolonged QTc¶	38 (13)	12 (12)	26 (13)	0.88
Sinus bradycardia	5 (1.7)	1 (1)	4 (2)	1.00
Sinus tachycardia	15 (5)	3 (3)	12 (6)	0.40
Sinus arrhythmia	6 (2)	1 (1)	5 (2.5)	0.67
ST change	1 (0.3)	0	1 (0.5)	1.00
Extrasystole	1 (0.3)	1 (1)	0	0
Vertigo†	16 (6)	12 (14)	4 (2)	< 0.001
Cough	4 (1.3)	3 (3)	1 (0.5)	0.10
Nausea†	11 (4)	10 (11)	1 (0.5)	< 0.001
Vomiting	10 (3.3)	5 (5)	5 (2.5)	0.30
Insomnia†	4 (1.5)	4 (4.5)	0	0.01
Fever	3 (1)	0	3 (1.5)	0.55
Lung abnormality#	24 (8)	7 (7)	17 (8)	0.70
Influenza	23 (8)	6 (6)	17 (8)	0.48
Weakness	3 (1)	2 (2)	1 (0.5)	0.25
Headache†	14 (5)	8 (9)	6 (3)	0.07
Tinnitus†	2 (0.7)	0	2 (1)	1.00
Labial herpes	1 (0.3)	1 (1)	0	0.32
Lymph node	1 (0.3)	1 (98)	0	0.32
Sore throat†	1 (0.4)	1 (1)	0	0.32
Convulsion	1 (0.3)	1 (1)	0	0.32
Diarrhea	4 (1.3)	2 (2)	2 (1)	0.60
Anorexia	4 (1.3)	4 (4)	0	0.01
Chickenpox	1 (0.3)	0	1 (0.5)	1.00
Impetigo	1 (0.3)	0	1 (0.5)	1.00
Pruritus	1 (0.3)	0	1 (0.5)	1.00
Dehydration	1 (0.3)	0	1 (0.5)	1.00
Splenomegaly	1 (0.3)	0	1 (0.5)	1.00
Abdominal pain†	3 (1)	1 (1)	2 (1)	1.00
Tonsilitis	1 (0.3)	1 (1)	0	0.32
Hypertension	2 (0.7)	0	2 (1)	1.00
Anemia	6 (2)	2 (2)	2 (2)	1.00
Renal stone	1 (0.3)	0	1 (0.5)	1.00
Mumps	1 (0.3)	0	1 (0.5)	1.00
Epistaxis	1 (0.3)	1 (1)	0	0.32
Upper respiratory infection	12 (4)	3 (3)	9 (4.5)	0.75

* Values are no. (%). Symptoms are given for only those 269 patients ≥ 3 years of age and able to answer questions about these symptoms. AE = adverse event.

† Denominators were 269 for all patients, 87 for the AM group, and 182 for the DP group.

‡ Cardiologic AE was any abnormality found on electrocardiographs.

§ Complete right bundle block. One patient had incomplete right bundle branch block (RBBB) on admission but complete RBBB at day 2 and incomplete RBBB on days 7, 28, and 63. Another patient had incomplete RBBB on day 0 but complete RBBB on days 2, 7, 28, and 63. These two patients were healthy and auscultation results of their hearts were normal.

¶ Prolonged QTc was defined as a QTc interval > 450 ms.

# Lung abnormalities were crepitations, rhonchi, or wheezing.

**Table 4:** Electrocardiographic analysis of patients in a study comparing AM and DP for treatment of *Plasmodium falciparum* malaria in Laos*

Variable	AM (n = 98)			DP (n = 202)		
	Baseline	Day 2	Day 7	Baseline	Day 2	Day 7
Heart rate, beats/min	115.9 (109.7–122.1)	96.1† (90.5–101.7)	96.9† (91.2– 102.7)	114.1 (109.9–118.3)	92.8† (88.5–97.1)	96.8† (92.8–100.7)
QTc Bazzet's, ms	422.3 (418.0–426.6)	426.5 (422.2–430.8)	427.4 (422.6–432.2)	421.2 (417.5–424.8)	430.7† (427.4–343.0)	422.5 (419.6–425.4)
PR interval, ms	131.3 (127.1–135.5)	140.8† (136.4–145.2)	137.5† (133.3–141.7)	135.4 (132.7–138.0)	140.9† (137.8–144.2)	139.2* (135.9–142.7)
QRS interval, ms	80.0 (77.6–82.4)	83.1† (80.6–85.6)	80.8 (78.1–83.5)	79.8 (77.9–81.2)	81.7† (79.9–83.5)	79.4 (77.7–81.2)
Correlation between QTc and RR interval	r = 0.001	r = −0.25	r = −0.25	r = −0.09	r = 0.13	r = −0.04
	*P* = 0.99	*P* = 0.01	*P* = 0.01	*P* = 0.23	*P* = 0.06	*P* = 0.57
QTc interval, ms§						
< 450	89 (92)	83 (86)	81 (85)	181 (90)	167 (85)	177 (90)
450–479	8 (8)	13 (14)	12 (13)	17 (8)	22 (11)	17 (9)
480–500	0	0	2 (2)	2 (1)	7 (3.5)	2 (1)
> 500	0	0	0	1 (0.5)	1 (0.5)	0
QTc lengthening, ms¶						
< 30	–	83 (87)	76 (81)	–	169 (86)	172 (88)
30–60	–	11 (12)	16 (17)	–	25 (13)	20 (10)
> 60	–	1 (1)	2 (2)	–	2 (1)	3 (1.5)

* Values are mean (95% confidence interval) unless otherwise indicated. AM = mefloquine plus artesunate for 3 days; DP = dihydroartemisinin plus piperaquine for 3 days.

† Significantly different compared with baseline (*P* ≤ 0.001).

§ Proportion of prolonged QTc reading by using Bazzet's correction.

¶ No. patients with QTc duration on days 2 and 7 by using Bazzet's correction.

**Table 5:** Review of efficacy or effectiveness of DP in 26 clinical trials (1,004 adults; 2,976 children; 2,030 not identified as children or adults; total = 6,010)*

Reference	Country	Comparator	Day of follow-up	No. patients		Cure rate (%)	No. deaths
				Adult	Child		
Asia							
37	China	0	28	60		96.7	Unknown
38	China	AL	28	51		100	Unknown
39	Cambodia	0	56	30	76	95.5	1 child (8-year old boy) at day 4
33	Cambodia	0	28	32	30	100	None reported
40	Cambodia	A + M	64	215		97.5	None reported
41	Vietnam	A + M	56	76		97.4	None reported
				40	283	98.7	None reported
42	Vietnam	AQ + P	28	84	0	94	None reported
43	Vietnam	A + AQ	42	34	15	100	None reported
44	Thailand	A + M	28	234	0	97	None reported
34	Thailand	A + M	28	134	0	98	None reported
			63	353		97	1 man at day 28 from gunshot
45	Thailand	A + M	28	120	0	99	None reported
46	Thailand	A + M	63	333		99.7	1 woman at < 24 hours after admission in the clinic (probably from severe malaria) unlikely caused by DP. 1 girl (13 years of age) at day 3 (unlikely caused by DP).
47	Thailand	AN + PPQ, A + M, and AL	28	82		98.8	None reported
35	Burma	A + M	42	327		99	One 11-year old died aparasitemic at day 21
11	Laos	A + M	42	36	74	100	A 2-year old boy died aparasitemic at day 37
48	Indonesia	A + AQ	42	92	76	96	None reported
49	Papua New Guinea	CQ + SP, A + SP, and AL	42	482		88	None reported
50	Vietnam	AN + PPQ	28	51		100	None reported
Africa							
28	Uganda	AL	63	0	351†	98	None reported
29	Burkina Faso, Kenya, Mozambique, Uganda, Zambia	AL	42	0	1,039	86	1 child (3 year-old girl) died 24 hours after treatment with DP. The most likely cause was sepsis or severe malaria.
51	Rwanda	A/SP + AQ	28	0	252	95.2	None reported
25	Uganda	AL	42	0	211	93	None reported
30	Kenya	AL	28	0	73	100	1 child (63 months of age) at day 13 unrelated to DP (probably caused by broncho-pneumonia)
27	Uganda	AL	42	0	215	98	None reported
26	Burkina Faso	AQ + SP and AL	42	0	187	98	None reported
South America							
52	Peru	A + M	63	168	94	98.4	None reported

* DP = dihydroartemisinin plus piperaquine for 3 days; AL = artemether-lumefantrine; A = artesunate; AN = artemisinin; M = mefloquine; AQ = atovaquone; P = proquanil; PPQ = piperaquine; CQ = chloroquine; SP = sulfadoxine-pyrimethamine.

† No. treatments rather than no. patients (longitudinal study).
